# The scaling of goals from cellular to anatomical homeostasis: an evolutionary simulation, experiment and analysis

**DOI:** 10.1098/rsfs.2022.0072

**Published:** 2023-04-14

**Authors:** Léo Pio-Lopez, Johanna Bischof, Jennifer V. LaPalme, Michael Levin

**Affiliations:** ^1^ Allen Discovery Center, Tufts University, Medford, MA, USA; ^2^ Euro-BioImaging ERIC Bio-Hub, EMBL, Germany; ^3^ University of Massachusetts Medical School, MA, USA; ^4^ Wyss Institute for Biologically Inspired Engineering, Harvard University, Boston, MA 02115, USA

**Keywords:** artificial embryogeny, active inference, artificial life, teleonomy, modelling, morphogenesis

## Abstract

Complex living agents consist of cells, which are themselves competent sub-agents navigating physiological and metabolic spaces. Behaviour science, evolutionary developmental biology and the field of machine intelligence all seek to understand the scaling of biological cognition: what enables individual cells to integrate their activities to result in the emergence of a novel, higher-level intelligence with large-scale goals and competencies that belong to it and not to its parts? Here, we report the results of simulations based on the TAME framework, which proposes that evolution pivoted the collective intelligence of cells during morphogenesis of the body into traditional behavioural intelligence by scaling up homeostatic competencies of cells in metabolic space. In this article, we created a minimal *in silico* system (two-dimensional neural cellular automata) and tested the hypothesis that evolutionary dynamics are sufficient for low-level setpoints of metabolic homeostasis in individual cells to scale up to tissue-level emergent behaviour. Our system showed the evolution of the much more complex setpoints of cell collectives (tissues) that solve a problem in morphospace: the organization of a body-wide positional information axis (the classic French flag problem in developmental biology). We found that these emergent morphogenetic agents exhibit a number of predicted features, including the use of stress propagation dynamics to achieve the target morphology as well as the ability to recover from perturbation (robustness) and long-term stability (even though neither of these was directly selected for). Moreover, we observed an unexpected behaviour of sudden remodelling long after the system stabilizes. We tested this prediction in a biological system—regenerating planaria—and observed a very similar phenomenon. We propose that this system is a first step towards a quantitative understanding of how evolution scales minimal goal-directed behaviour (homeostatic loops) into higher-level problem-solving agents in morphogenetic and other spaces.

## 1. Introduction

How does the complex anatomical structure of the body form reliably from the activity of individual cells? More than emergent morphogenesis, many organisms are able to reach the same specific target morphology despite surgical injury, changes of cell size or number, organ rearrangement, genetic defects and many other insults [1]. How does the collective decision-making of cell groups enable regeneration, regulative, development, metamorphosis and cancer suppression? Answers to these questions are critical to many fields, including evolutionary developmental biology, cell biology and complexity science [2,3]. Beyond their importance for basic science, the ability to control the collective behaviour of cells toward specific anatomical shapes has massive implications for transformative biomedicine. How can we best control morphogenetic systems composed of several levels of organization from cell to tissue, to achieve desired outcomes in regenerative medicine and synthetic bioengineering [4]?

All of these questions raise the problem of understanding how the competencies of cells relate to those of the emergent tissue-level swarm, and what evolutionary dynamics enable the shift between those states to occur. No individual cell knows what a finger is or how many a salamander is supposed to have, and yet when amputated, the collective will actively build until precisely the right number, size, shape and position of the fingers is restored [5]. What is the origin of the dynamics that enable a cellular collective to work toward large-scale anatomical target states that are too complex and big for individual cells to represent?

Some frameworks, motivated by cybernetics and behaviour science, seek a deep symmetry among homeostatic behaviour across scales and material implementations [6,7]. It has been suggested [8] that evolution pivoted some of the same strategies for navigating problem spaces across metabolic, anatomical and behavioural domains. For example (reviewed in [9,10]), the same mechanisms using ion channels, electrical synapses and computations via bioelectrical networks underlie problem-solving in physiological space by bacterial communities [11,12] and plants [13,14]; navigation of morphospace by a wide range of embryonic and regenerative systems [15–17]; and classical behaviour by organisms with neurons and brains [18,19].

Traditional behaviour science focuses on the cognitive capacities of an ‘individual’—memory, perception, learning, anticipation, decision-making, goal-directed behaviour—and makes two assumptions. First, the body is usually conceived as a fixed structure, following the mainstream paradigm that the genome codes for specific bodyplans. Second, brain structure is thought of as stable—the individuality of its neuronal cells is gone for good. Our primary experience is that of a centralized, coherent self. For organisms like caterpillar, which undergo metamorphosis, with drastic changes of body, brain and behaviour, studies usually focus on their separate phases of life. The transitional states are usually not studied [20].

However, all intelligences are collective intelligences, they are made of parts that are themselves biological agents. Regenerative biology and the development of new organisms like biobots [21] reveal that studies of cognition in intact bodies present just a narrow slice of a much bigger picture: that of the multi-scale interface between body and mind. Indeed, cognition is tightly linked to the physical structure of the body, from the anatomical level to the molecular and bioelectric levels that store its information [20]. And structure and function are both highly plastic. Cognition continues to function despite important changes to the body/brain and the corresponding modification of its information at the cellular, molecular, or bioelectric level [22].

In the age of regenerative medicine, and of hybridization between evolved and designed subsystems [23], it seems essential to understand how mind and body coevolve [24], in order to explore the origin of multicellularity and the scaling of basal cognition of individual cells into larger organisms. The algorithms that allow cells to perform goal-directed decision-making and the transition to larger selves are still poorly understood [20,25]. To integrate and advance the fields of developmental/evolutionary biology, synthetic bioengineering, artificial intelligence and cognitive science, it is fundamental to begin to develop computational frameworks that allow us to ask how cognitive capacities arise in agents made up of parts, and how these emergent cognitive agents scale up during evolution.

In the scale-free cognition framework [20], we rely on ideas from control and information theory to identify multi-scale information processing principles. That theoretical framework proposed that the scaling of homeostatic behaviour in morphogenetic space is the direct result of group dynamics of homeostatic subunits in metabolic space [20]. The work we present here is a quantitative *in silico* embodiment of that theory, generating testable predictions. It provides, to our knowledge, the first exploration of the emergent morphogenetic consequences of a scaling that does not treat cells as simple automata following static rules, but instead as entities with information-processing homeostatic capacity. Earlier studies showed that individuals can be outperformed by collectives [26], and that a collective’s overall performance is dependent on several characteristics including the organizational or network structure [27], the information aggregation and communication system among individuals, and the diversity of members [28]. These studies focused mainly on human networks and the associated wisdom of the crowd. We focus here on morphogenetic systems.

The mechanistic and algorithmic invariance between scales of organization has enabled numerous tools and approaches from neuroscience to be ported to developmental biology [4,29,30] resulting in novel capabilities in regeneration, cancer reprogramming, and repair of birth defects. To move this field forward, and to better understand the role of evolution, it is essential to develop quantitative, generative models that reveal what dynamics are sufficient for the scaling of competencies and their generalization to new problem spaces. Thus, we sought to create a minimal *in silico* system in which we could observe whether, and how, evolutionary dynamics could drive the shift from cell-level metabolic goals to the much larger, body-wide goal of patterning a positional information axis. The scope of this work applies to any organism capable of axial patterning including not only metazoans but also plants, corals and sponges [31–33]. Goals are here defined from the perspective of a cybernetic approach, focused on error minimization with respect to a specified homeostatic setpoint [34,35]. Here, we present the details and analysis of a simple artificial embryogeny system (two-dimensional cellular automata with each cell’s behaviour controlled by an evolutionary artificial neural network) which we initialize with cell-level physiological homeostasis and observe the emergence of competence to reach a target morphology (solving the classic French flag problem [36]) in an entirely new problem domain: anatomical morphospace. While a number of models have been proposed for solving the French flag problem [37–40], our model uniquely shows how the capability to robustly self-organize a spatial axis at the tissue level emerges from unicellular metabolic competencies (cellular homeostasis in the absence of local instruction). In this work, we used the French flag problem as a measure of morphogenetic competency to flesh out a specific theory of how collective intelligence scales up across problem spaces in evolution. Our data show that evolutionary forces drive the emergence of several higher-level competencies, including error-minimization to reach an anatomical goal state and robustness to perturbation. This multi-scale homeostasis is driven by shared (non-local) stress dynamics and biologically plausible cell–cell interactions. Our analysis reveals how minimal low-level metabolic requirements can scale up into morphogenetic competency.

## 2. Foundation for the model

Our model is based on the following background assumptions as defined in detail in the TAME framework [7]. The first is that individual cells already have the competency to stay alive via homeostatic pursuit of metabolic resources. This is a minimal requirement for all living forms (already present in microbes, both unicellular and multicellular), but we assume its presence—we are not modelling the origin of life or the initial formation of the very first reproductive unit, but rather seeking to understand the collective behaviour of subunits that were once independent organisms themselves.

Second, we model two scales: a developmental phase where cells are alive and interact with each other, and an evolutionary wrapper which changes the frequency of different behavioural policies of the cells based on the fitness of the collective (how well the resulting ‘embryo’ matches a very simple criterion: having a single, three-valued positional information axis).

Third, our use of the term ‘goal’ is not meant in any kind of meta-cognitive, self-aware complex sense. Goals here are synonymous with the cybernetic approach [34,35], in which certain systems can expend effort to reduce error from a specified homeostatic setpoint. Our aim is to show how such goals can scale in size and arise spontaneously in new problem spaces under evolutionary selection, given very minimal and realistic assumptions about the properties of the components.

Finally, while we do not explicitly invoke bioelectric signalling mechanisms, our cell interaction model is quite compatible with that mode of communication (spelled out in detail in [20]). We do provide the cells with gap junctions (electrochemical synapses [41,42]), through which they can share state signals. We also include a mechanism for stress signals (initially, intracellular detectors of out-of-homeostasis states) to be propagated outward from cells, experiencing geometric frustration (a measure of how much the neighbours of a cell share the same state) as predicted by the TAME framework [7].

## 3. Related work: *in silico* embryogeny

**Figure 1.**
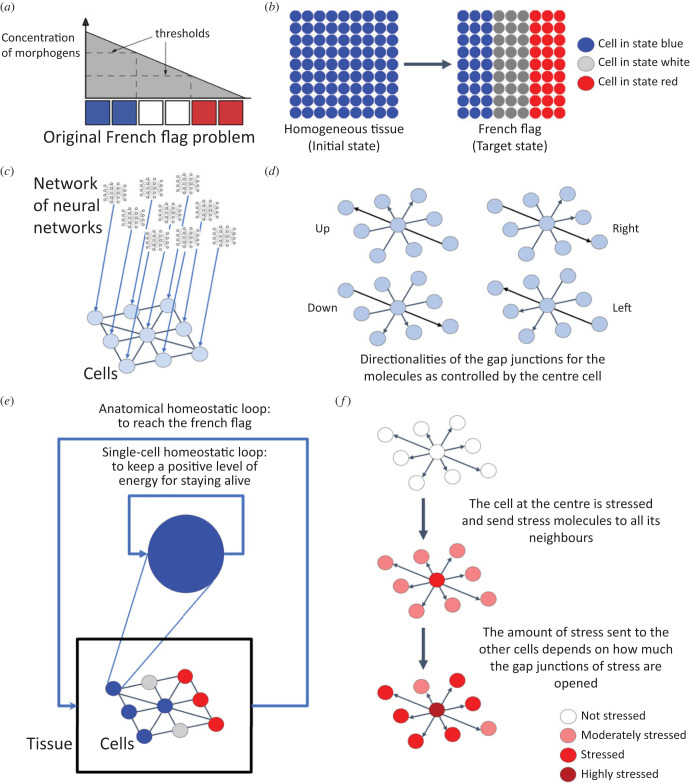
Description of the general scheme of the evolutionary system. (*a*) Description of the French flag problem. The concentration of morphogen triggers a particular gene expression leading to three different states: blue, white or red. (*b*) Description of the virtual environment. At left, the tissue is uniformly initialized. At right, the tissue has reached the French flag. (*c*) Each cell is connected to its neighbours and can exchange morphogen molecules via gap junctions. The behaviour of each cell is controlled by its neural network. (*d*) Directionalities of the gap junctions as controlled by the neural network embedded in the cells. (*e*) Two homeostatic loops have been implemented: a homeostatic single-cell loop, in which each cell has to maintain a positive level of energy to stay alive; and an anatomical homeostatic loop, in which all cells receive energy depending on how closely the collective (or the tissue) approaches the anatomical goal. (*f*) Spread of the stress molecule to neighbouring cells as controlled by the ANN inside each cell. Alternatively, each cell can send an anxiolytic compound to all its neighbours in the same way.

Multicellular morphogenetic algorithms or set of built-in behavioural and signalling policies that allow cells to cooperate and compete to reliably construct complex body pattern are still incompletely understood [20,25]. One relevant approach is amorphous computing, which refers to systems of many identical simple processors that have limited computational power and that interact locally. Typically, such systems have a more irregular structure in contrast to the regular structure of cell networks as they are used in our study [43]. Several authors have investigated pattern formation in developmental biology using the same task we use in this article, the French flag problem. Herman and Liu solved it through the simulation of linear iterative arrays of cells [44]. Several other models have been developed to resolve the same problem, including a reaction–diffusion model, use of computational primitives and genetic programming [37–40]. Miller evolved a development program that follows a predefined grammar that connects developmental encodings to genetic programming. He used a feed-forward Boolean circuit to implement a cell program [38]. Chavoya and Duthen used a genetic algorithm to evolve cellular automata that produced different two-dimensional and three-dimensional shapes [45] and evolved an artificial regulatory network (ARN) for cell pattern generation, resolving the French flag problem [46]. While others have simulated evolutionary growth of neural network-controlled cellular automata with hardwired mechanistic rules, this is the first exploration of the emergent consequences of evolutionary scaling based on dynamic interactions of cells with information-processing homeostatic capacity. The combination of neural networks and cellular automata which grow under evolutionary control is described in the work of Elmenreich & Mordvintsev [47,48]. However, their neural cellular automata do not include any cellular signalling or any use of stress capable of linking the different homeostatic levels for the scaling of goals (metabolic in individual cells and morphogenetic for the tissue). A key difference in our work is that the artificial neural network (ANN) inside each cell here models the behaviour of a gene-regulatory network and can control gap junctions to deliver the morphogen and the ability to reduce stress (tendency to reduce geometric frustration) to the other cells (figure 1).

Past studies have also investigated the multi-scale interface of body and mind, notably with ‘morphological computation’ in artificial life and soft evolutionary robotics [49–53]. These studies model and exploit the fact that brains, like other developing organs, are not hardwired but are able to ascertain the structure of the body and adjust their functional programs accordingly. Similar to scale-free cognition, morphological computation is about connecting the body and cognition—showing how optimal control policies span different scales, mechanisms and problem spaces. However, to our knowledge, no prior model has explicitly investigated how cell-level metabolic competencies scale up into tissue-level morphological ones.

## 4. Methods

### 4.1. An *in silico* evolutionary system for the study of transitions from single-cell homeostasis to anatomical homeostasis of emergent tissue-level axial patterning

#### 4.1.1. The general scheme of the evolutionary simulation

##### 4.1.1.1. General system

We developed an evolutionary simulation system composed of cells that forms a tissue on a two-dimensional grid (figure 1). The system is an evolutionary, agent-based, spatialized model that uses two main time-scale loops, with different temporal scales. The outside loop is an evolutionary (phylogenetic) long time scale where genomes are mutated (here the genome is the one corresponding to the evolutionary algorithm and it generates the ANN controlling each cell in the tissue and agents are selected). The inner loop is a short time-scale ontogenetic loop that constructs each agent, simulates development, and then tests the phenotype for fitness. Each agent is one cell, that encodes some very simple metabolic processes and interacts with the other cells.

We integrated a homeostatic loop that enables it to optimize for the levels of energy (we are not trying to model the origin of life itself—we start with cells that can keep themselves alive via basic homeostasis, e.g cells that need to keep a level of energy superior to 0). We allow cells to attach to each other using gap junctions, to send intracellular signals. These signals, and their properties—such as speed of spread, ability to propagate gap junctions, gating properties of gap junctions based on signal and cell state etc.—are all controlled by the ANN inside each cell through evolution. Cells do not know the origin of a given signal once it is inside the cell; it has been suggested that this ‘wiping property’ enables a kind of shared memory that is essential for the scaling of small agents into larger intelligences [7]. The cells have a minimal memory of the past and have access to their stress and energy levels, and their states, at time *t* and *t* − 1 (see algorithm 1).

The general approach is related to cellular neural networks [54] and more particularly to the growing neural cellular automata [48] where each cell has access to the states of its neighbours and contains a neural network to drive actions (neural networks are simply a convenient formalism for encoding cell behavioural properties, equal in power to gene-regulatory networks [55]). In our case, the number of cells is fixed and does not grow with time and the tissue formed by those cells has borders (i.e. it is not a torus). The order of execution of cells is shuffled at each step.

##### 4.1.1.2. Cell description

Each cell contains an ANN (representing the computational functions carried out by gene-regulatory networks and pathways operating within cells [55,56]) which controls the opening and closure of gap junctions in four directions: up, down, left, right. ANNs are commonly used to model genetic regulatory networks [57]; in that line of research, the ANN inside each cell can be interpreted as a genetic regulatory network controlling the metabolic processes. Each cell has one type of morphogen molecules that can go through the gap junctions depending on their openings and that will trigger different kinds of genomic expression leading to three different cell states: blue, white, or red (see figure 1*a*). These states depend only on the level of morphogen inside the cells (red if the level of morphogen is between 0 and 5, white if between 5 and 10, blue if greater than 10). We also imposed an energy cost on state change. Each cell also has a stress molecule and an antistress counterpart that can be sent to its neighbours (see algorithm 2). To summarize, each cell can contain the following: the energy state, necessary for survival and for state change, morphogens that can go through gap junctions to define the cell state; and stress and stress-reduction molecules, which can also move between cells via gap junctions, and which define the stress state.

Our model does not enforce the use of the stress system: it is completely evolved since the number of stress and stress-reduction molecules to be transferred is chosen by the embedded neural network. In addition, evolution can decide to make the stress molecule instructive or not. We have chosen to refer to it as the stress molecule but, before evolution, it is just another communication system for cell signalling that does not directly affect the cell state (see §4.1.1.5). The neural network, which is identical for each cell, has 11 inputs: the internal levels of morphogens, energy at time *t*, energy at time *t* − 1, stress at time *t*, stress at time *t* − 1, the internal state at time *t* and *t* − 1, the size of the collective the cell is part of (number of cells of the same state connected by opened gap junctions in the tissue), the error between the size of the collective the cell is part of and the target size in percentage—e.g. the size of one stripe—(|100 − [size of sub-collective/(total number of cells in the tissue/3 × 100)]|), the perception of the cell neighbourhood (geometrical frustration or how similar the cell is to its neighbours) and a bias set at 0.5. The four outputs of the ANN are: the number of morphogen molecules to send to neighbours, the number of stress molecules to send to neighbours and to be applied to the cell itself, the number of stress-reduction molecules to send to neighbours and to be applied to the cell itself, and the opening of gap junctions in the four directions. The level of stress of one cell is bounded between 0 and 100.

##### 4.1.1.3. Learning task

The learning task is the French flag problem [58,59] (see figure 1*b*). It was originally developed by Wolpert [58] to formalize discussions about how spatial gradients might specify patterns of cell fates in a tissue.

##### 4.1.1.4. From local to anatomical homeostasis

We tied the single-cell and the anatomical homeostatic loops. Each cell has only one goal—to survive—and that corresponds to being in the appropriate state in order to receive energy (with the other members of the collective). On the other hand, the collective/tissue has a morphogenetic goal, which is to reach the French flag.
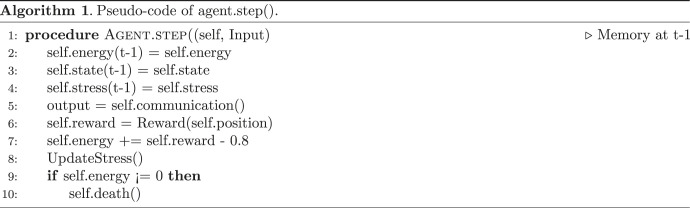
 
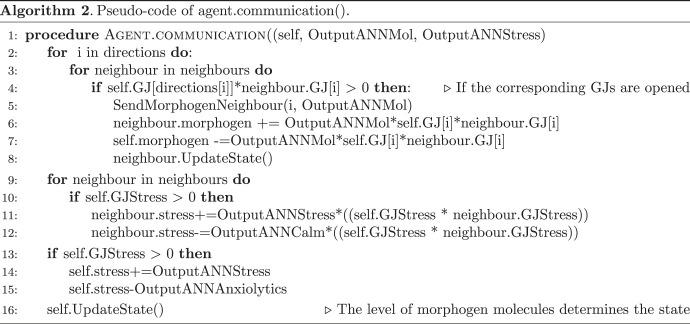


At each step, each cell receives a reward in the form of an amount of energy which is proportional to how close its corresponding sub-collective (corresponding to one stripe and therefore bigger that the immediate neighbourhood of one cell) is to reaching the appropriate anatomical goal. Each cell therefore has a reward uncertainty, because the reward depends not only on that cell’s own behaviour but also on the behaviour of the (sub)collective; in other words, the reward is affected by the decisions of distant cells. Therefore, the environment of the cells is of high uncertainty. In a sense, this scheme can be understood as a problem of multi-agent reinforcement learning under reward uncertainty.

Goal-directed systems have the ability to focus on relevant information and ignore distracting information. To do so, they rely on selective attention and/or interference suppression. Selective attention would rely on top-down biasing mechanisms as proposed by Desimone & Duncan [60]. In our case, the top-down biasing mechanism is represented by the reward in energy that ties the two homeostatic levels. We also imposed an energy cost for communication and state changes (respectively, of 0.8 and 0.25 per step).

##### 4.1.1.5. Stress system

Stress can be defined as three related concepts—the external and internal stimuli that cause stress, the emergency physiological and behavioural responses activated in response to those stimuli, and the pathological consequences of over-stimulation of the emergency responses [61,62]. Here, we implemented a communication system that enables diffusion of a stress molecule through the tissue to allow other cells to feel stress that was not caused by their own internal state. When the ANN of a cell makes the decision to send stress, that cell will diffuse a molecule to its neighbours, equally increasing its own stress level and that of its neighbours. In the same manner, the ANN can also cause the cell to send a stress-reduction molecule (functionally equivalent to an anxiolytic intervention) to the neighbouring cells that will decrease the cell stress levels (figure 1*f*). Importantly, whether or not (and how) the stress system is used to solve the patterning problem is not determined in the code. Evolution can produce agents that do or do not use this set of signals as a communication system. In other words, before evolution, what we call the stress system here is just another communication system in the tissue where a specific cell signalling will increase (or decrease) the level of a molecule inside the cell. This cell signalling works exactly like the morphogen communication system, except that it does not have any effect on the cell state. Evolution can choose whether or not to add an ‘interpreter’ to these specific molecules (e.g. the ones we defined in this section as stress and stress-reduction molecules) to make their levels informative for the cells or by triggering a specific behaviour (like biological stress).

##### 4.1.1.6. Evolutionary algorithm

We use ES-HyperNEAT [63] to simulate the evolutionary cycle. ES-HyperNEAT (evolving substrates HyperNEAT) is an extension of the original HyperNEAT method for evolving large-scale artificial neural networks using the NEAT method (neuroevolution of augmenting topologies). HyperNEAT evolves large-scale neural networks using the geometric regularities of the task domain. It uses compositional pattern producing networks (CPPNs) to generate the structure of the ANN. ES-HyperNEAT allows the positions and number of the neurons to be evolved instead of being defined before evolution as in HyperNEAT [63]. The fitness function is the percentage of good states the tissue has:

4.1 fitness=(number of good states/number of cells)×100.

The whole system has been coded in Python programming language, using the agent-based modelling framework Mesa [64] and the MultiNEAT package. It is freely available on https://github.com/LPioL/scalefreecognition.

##### 4.1.1.7. Parameters

All experiments in this article used the same parameters and have been repeated over 20 runs. For each evolution, we used 250 generations, a population size of 350 individuals, a division and variance thresholds of 0.03. The energy cost for state change is 0.25 and at each step, cells lose 0.8 in energy. The minimum and maximum number of species are, respectively, 5 and 15. The number of generations without improvement (stagnation) allowed for a species is 10. The depth for the neural net is four hidden layers (increasing the number of hidden layers to five did not change the general fitness score) and three for the quadtree.

The available CPPN activation functions for the French flag task domain were sigmoid, Gaussian, linear, sine and step. The band-pruning threshold for all ES-HyperNEAT experiments was set to 0.3. The bias value for the CPPN queries is −1. The cells starts with energy levels initialized at 70. The energy cost of communication is set at 0.8. We also applied an energy cost of −0.25 to the state change for all cells.

#### 4.1.2. Information-theoretic analysis

Information theory [65] is a very useful tool to understand the information dynamics in complex systems. We used two information-theoretic measures to analyse the information dynamics of the results: active information storage (AIS) [66] and transfer entropy [67].

##### 4.1.2.1. Active information storage

The amount of information in the past of one agent that is relevant to predict its future state is defined as the information storage. In this article, we focus on the AIS component, which is the stored information that is currently in use for computing the next state of the agent [66]. Formally, the AIS of an agent *Q* is defined as the local (or unaveraged) mutual information between its semi-infinite past $q_{n}^{\left(k\right)}$ as *k* → ∞ and its next state *q*_*n*+1_ at time step *n* + 1:

4.2 $$a_{Q}(n+1)=\lim_{k\to \infty }\log_{2}\frac{\;p(q_{n}^{\left(k\right)},q_{n+1})}{p(q_{n}^{\left(k\right)})p(q_{n+1})}.$$

*a*_*Q*_(*n*, *k*) represents an approximation of history length *k*. The average over time (or equivalently weighted by the distribution of $\left(q_{n}^{\left(k\right)},q_{n+1})\right)$: *A*_*Q*_(*k*) = 〈*a*_*Q*_(*n*, *k*)〉. With AIS, an agent can store information regardless of whether it is causally connected with itself [66].

In this article, we compute the local AIS over the states of the cells.

##### 4.1.2.2. Transfer entropy

Transfer entropy is the information provided by the agent source about the destination’s next state that was not contained in the past of the destination agent. In this article, we use the local transfer entropy introduced by Lizier [66]. The local transfer entropy from a source agent *Z* to a destination agent *Q* is the local mutual information between the previous state of the source *z*_*n*_ and the next state of the destination agent *q*_*n*+1_, conditioned on the semi-infinite past of the destination $q_{n}^{\left(k\right)}$ (as *k* → ∞):

4.3 $$t_{Z\to Q}(n+1)=\lim_{k\to \infty }\log_{2}\frac{\;p(q_{n+1}|q_{n}^{\left(k\right)},z_{n})}{p(q_{n+1}|q_{n}^{\left(k\right)})}.$$

Transfer entropy *T*_*Q*_(*n*, *k*) is the (time or distribution) average: *T*_*Q*_(*k*) = 〈*t*_*Q*_(*n*, *k*)〉 and *t*_*Q*_(*n*, *k*) represents an approximation of history length *k*. While mutual information measures correlation only, the transfer entropy measures a directed and dynamic flow of information in the network of agents.

For the analysis of our information dynamics, we computed a different kind of transfer entropy: the transfer entropy from stress (by discretizing it by chunks of 10) to the states of the neighbours (blue, white, red). We computed the local transfer entropy pairwise with all the neighbours of one cell and averaged it. In the same manner, we computed the transfer entropy from the energy state to the state of the cells and conversely the transfer entropy from the state of the cells to the energy state.

We also defined the transfer entropy from one stripe to another as the sum of all transfer entropy of one cell of one stripe to all cells of the other stripes computed pairwise on the time series of the internal states. Formally, we define the averaged transfer entropy over a range of source–destination pairs in the spatial locations of different stripes *S*_1_ and *S*_2_:

4.4 $$t_{S_{1}\to S_{2}}=\sum_{Z_{i}\in S_{1}}^{\;}\sum_{Q_{\;j}\in S_{2}}^{\;}t_{Z_{i}\to Q_{i}}.$$

The transfer entropy defined for specific subsets of tissue processes is useful in considering distributed communications across agents with specific roles.

### 4.2. Method for the planaria experiment on repatterning

#### 4.2.1. Colony maintenance

*Dugesia japonica* were maintained in Poland Spring water at 20°C, fed calf liver paste once a week and cleaned twice a week, as described in [68]. Animals were starved for one week prior to usage in amputation and pharmacological treatment experiments and were not fed for the duration of all experiments.

#### 4.2.2. Animal manipulation

Cutting of planaria was performed on a cooling plate using scalpel fragments. For generating headless animals, pre-tail fragments were cut by placing a cut at the pharynx opening and another cut narrowly above the tail tip. For tracking tail regeneration, animals were cut halfway between the head and tail. For tracking regeneration along the anterior/posterior axis, animals were either decapitated narrowly by cutting just below the auricles, cut halfway between the base of the head and the top of the pharynx, or cut directly at the top of the pharynx.

#### 4.2.3. Pharmacological treatments

Headless worms were produced through transient pharmacological treatment of freshly cut pre-tail fragments in 18 μM U0126 (a well-known blocker of ERK/MAP kinase [69]) dissolved in DMSO (Sigma). No more than 40 fragments were treated per 10 cm Petri dish. Fragments were incubated in U0126 for 3 days at 20°C before washing out the drug solution. At 14 days post-amputation, the fragments were scored for regenerative phenotype.

#### 4.2.4. Scoring of repatterned phenotypes

Headless animals were maintained in individual wells of 12-well plates and their phenotypes were scored weekly for the duration of the experiment. Any significant changes in morphology, such as head regeneration, fissioning or ectopic tissue development, were noted. Time of repatterning was determined once one eye was visible in the forming head structure. Worms were labelled as ‘Polarity Flip’ when a headless worm fissioned and a head regenerated at the posterior wound site. Worms were labelled as ‘Dorsal/Ventral repatterning’ when an outgrowth of tissue occurred in the dorsal/ventral (D/V) plane of the headless worm. Worms were labelled as ‘Lateral growth’ when significant changes in morphology occurred with growth on the lateral area of the animal, resulting in stable morphology which did not produce a head.

## 5. Computational results

We analysed a number of experiments performed with this system, tracking key physiological parameters over time in each experiment (see figures), including gap junctional communication, stress levels and cell types as a function of position. We focused on the most biological evolved tissue: the one where stress emerged during evolution as an instructive signal and increases and decreases as a function of the homeostasis of the tissue.

### 5.1. The tissue minimizes error for reaching the target morphology

We first tested the ability of the cellular collective to solve the French flag problem, that is, to organize the two-dimensional tissue into a one-dimensional axis of positional information with respect to cell type identity. Each cell received energy according to is location on the tissue, and its energy reward was proportional to how well the other cells of one stripe of the French flag were resolving the French flag pattern (in other words, evolution gives partial credit for imperfect primary axial patterning, selecting for embryos with optimal morphogenesis). The cell colours in all figures represent cell fate, as in the original definition of the problem in which an embryo must spontaneously pattern itself into three ordered regions of cell fate [58]. All cells started in the blue state (homogeneous tissue corresponding to an un-patterned nascent blastoderm). To solve the problem, cells had to dynamically cooperate with, and send the appropriate signals to their neighbours. The fitness function was computed for 100 steps, defining the time course of development in this virtual embryo.

**Figure 2.**
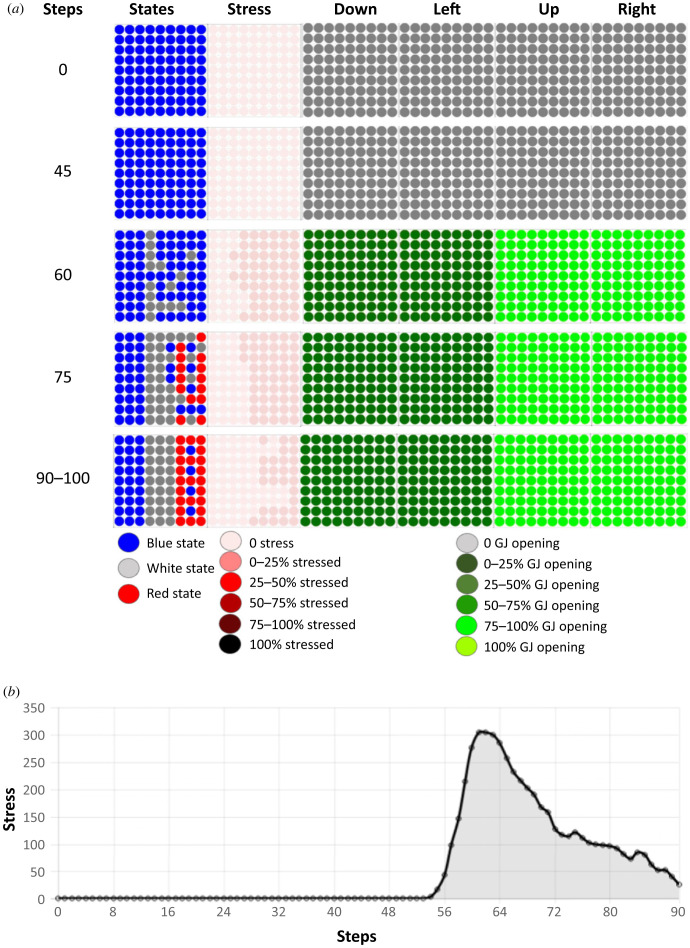
Dynamics of resolution of the French flag problem. (*a*) Temporal dynamics of changes in cell state, stress and gap junction status during problem resolution. The cells resolve the French flag problem almost entirely by the 90-step timepoint. The dynamics of the resolution is represented by five time steps taken at steps 0, 45, 60, 75 and 90–100 of the simulation. These five time steps are indicated in the leftmost column, and the remaining columns represent (from left to right): the states of the cells in the tissue (blue, white, red), the use of the stress system on a colorimetric scale (the darker the red, the more the system is stressed), and the percentages of open gap junctions in four directions (down, left, up and right), again using a colorimetric scale. Grey represents closed gap junctions and the green scale denotes the range of opening: the darker the green, the smaller the percentage of open gap junctions. The stress system is used throughout the resolution of the French flag problem. (*b*) Amount of stress in the tissue during the resolution of the French flag problem. The stress system is used all through the resolution of the French flag problem. The amount of stress increases around step 55 and decreases rapidly after about step 64, approaching zero when the target morphology is reached around step 90.

We observed that the ANN inside each cell evolved, and that this system was able to solve the problem (figure 2). A typical tissue behaviour had the following features. First, the regions corresponding to future white and red stripes began to be stressed and then it was mainly the red stripe that was stressed. All gap junctions were open, the upper and right gap junctions were fully open and the low and left gap junctions were half open. The gap junction states weighted the flow of molecules so that the streams from right to left and left to right were equal as was also the case for the streams in the vertical axis. The diffusion of morphogen molecules from left to right occurred because the cells of the blue stripes acted as a reservoir of morphogens and the cells decided to send more or fewer of these molecules to their neighbours depending on the dynamics.

In the representative example we show here, the stress increased at 55 steps and it then decreased as the French flag morphogenetic problem was resolved. At 90 steps, the tissue reached 95.1% of the French flag target morphology, and there were four remaining blue cells in the red stripe (an almost perfect solution). The same result was seen over 20 repeat runs, solving the problem in 94.4±0.84% of over 20 runs. The sharp change in morphogenetic activity around step 55 appears because the cells had learned to be stressed at a specific energy level. This converts continuous metrics to sharp discontinuities—the perception and decision-making that cells have learned over their history. This is demonstrated by our finding that when the stress level at initialization is increased, the spike in stress is delayed proportionally (see electronic supplementary material, figure S1).

Thus, the tissue learned to minimize error between its current state and the target morphology during its lifetime in order to stay alive; by doing so, the tissue—starting from a homogeneous state—resolved the French flag problem. It seems to work less well with random initializations, suggesting that robust development is optimized when cells start with a muted, homogeneous ‘baseline’ state, not highly diverse starting states, which is indeed what is observed in real development, where embryos do not begin as a collection of randomly differentiated cells, but as a homogeneous mass of undifferentiated ones (see electronic supplementary material, figure S3). We conclude that this minimal system shows how cell behaviours (that can be tuned by evolution or learning) enable the collective to harness individual metabolic homeostatic loops (the pursuit of energy) towards a global patterning goal.

### 5.2. The tissue is robust to perturbation

The cells had learned error-minimization in order to reach the anatomical goal, but did they follow a hardwired plan that could only enable a feed-forward emergent pattern? Or had they in fact acquired an ability for homeostasis that would allow the tissue to reach the target morphology even in the presence of perturbations beyond the *ab initio* morphogenesis?

**Figure 3.**
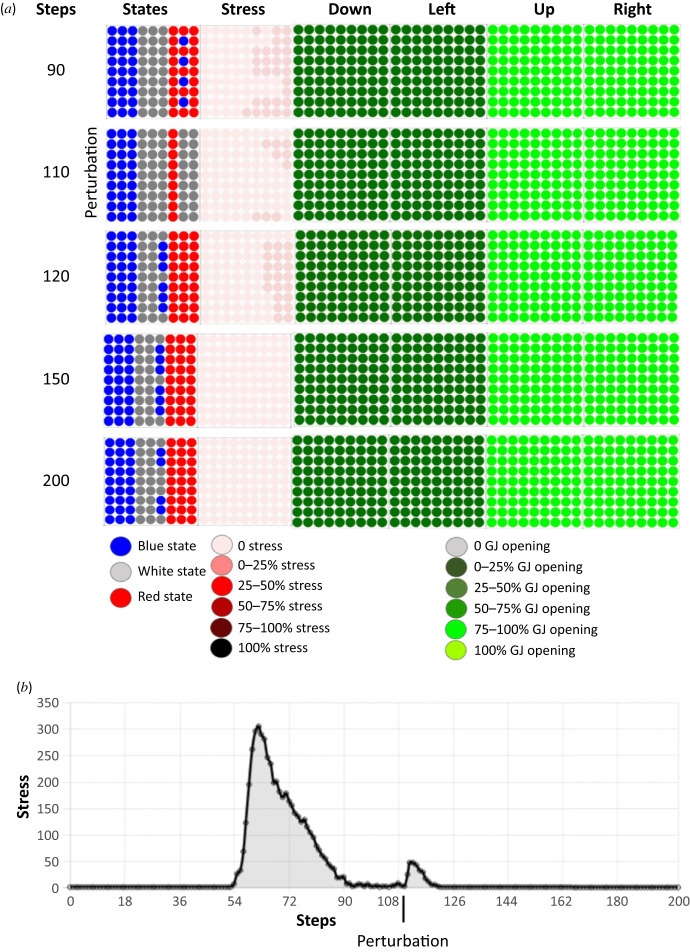
Dynamics of the resolution of the French flag problem after an induced perturbation. (*a*) Tissue dynamics are represented by five time points taken at steps 90, 110, 120, 150 and 200. These five time steps are indicated in the leftmost column, and the remaining columns represent (from left to right): the states of the cells in the tissue (blue, white, red), the use of the stress system on a colorimetric scale (the darker the red, the more the system is stressed), and the percentages of open gap junctions in four directions (down, left, up and right), again using a colorimetric scale. Grey represents closed gap junctions and the green scale denotes the range of opening: the darker the green, the smaller the percentage of open gap junctions. As in figure 2, the problem is nearly perfectly resolved by step 90, but the system is then experimentally perturbed at step 110. Following perturbation, the cells resolve the problem almost entirely by step 200. (*b*) Amount of stress inside the tissue during resolution of the French flag problem following perturbation. The stress system is active throughout the resolution of the French flag problem and it increases and decreases with pattern resolution following the perturbation.

To determine the degree of plasticity of this process, in this next set of simulations, we perturbed the tissue by artificially changing at 110 steps the states of the last two columns of the red stripe to white cells. The cells had never been evolved on more than 100 steps. However, after the perturbation at 110 steps, the tissue corrected the red stripe in 10 steps and a few blue cells appeared in the white stripe (figure 3). The stress increased and decreased in parallel to the perturbation and its resolution, as it did to resolve the French flag problem. This capacity is similar to that observed in biological systems, many of which are able to regain normal morphology despite a wide range of perturbations [1]. The system tried to get rid of the remaining blue cells left in the white stripe. At 200 steps, the tissue reached 95.1% of the French flag. The dynamics of the gap junctions stayed the same as during the French flag resolution as seen in figure 2 without perturbation.

We also examined the effect of changing the size of the perturbation domain (see electronic supplementary material, figure S2). The tissue can adapt sufficiently for survival following a range of smaller perturbations (from two cells to two columns within a stripe) made at step 110. However, it was unable to recover from a much larger perturbed region (e.g. converting the entire red stripe into a white one). In this case, it attempted to adapt but dies at 200 steps. For smaller perturbations, the tissue reached a French flag compatible with survival. Interestingly, biological data support this surprising result: in the case of transformation of normal melanocytes in tadpoles to body-wide melanoma [70], and in the case of transplantation of healthy tissue into a deformed tadpole to repair its brain development [71], a very few cells are necessary to effect system-wide morphogenetic change.

Thus, we conclude that even though we did not specifically reward for anything other than the single self-organizing property, the cells were able to repair to the target setpoint from multiple starting configurations. In this sense, the tissue is excitable as a perturbation will spread and change the morphology.

### 5.3. The tissue maintains allostasis in adulthood

**Figure 4.**
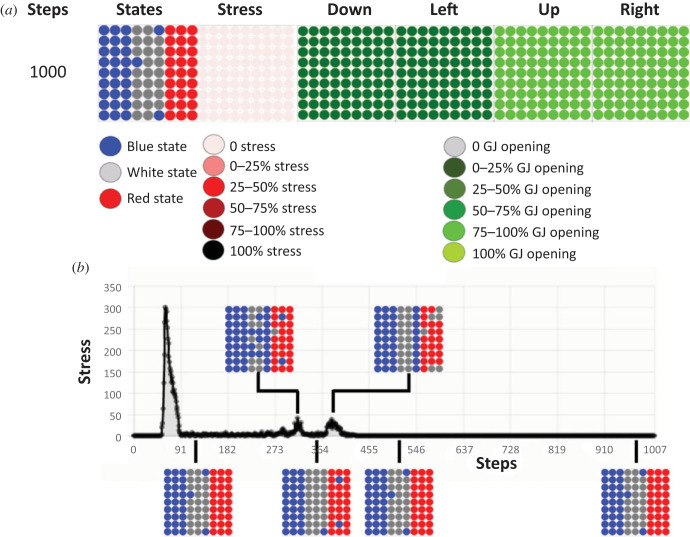
Long-term survival of the tissue and stress. The simulation was run for 1000 steps. (*a*) State of the tissue at 1000 steps. The state of the tissue is represented at step 1000 of the dynamics of the tissue. Simulation time is indicated in the leftmost column, and the remaining columns represent (from left to right): the states of the cells in the tissue (blue, white, red), the use of the stress system on a colorimetric scale (the darker the red, the more the system is stressed), and the percentages of open gap junctions in four directions (down, left, up and right), again using a colorimetric scale. Grey represents closed gap junctions and the green scale denotes the range of opening: the darker the green, the smaller the percentage of open gap junctions. At 1000 steps, the tissue reached 96.3% of the French flag target morphology. (*b*) Amount of stress inside the tissue during its lifetime. Stress increased and decreased as the tissue encountered and resolved deviations from the target morphology due to its intrinsic dynamics.

We discovered that the tissue can resolve the French flag problem and is robust to perturbation, but does the tissue reach long-term survival and maintenance of an adult phase? To answer this question, we performed simulations running for 1000 steps. We observed that the tissue maintained its morphology during the whole lifetime. In addition, at 1000 steps, the morphology was even better than in the developmental phase, with the tissue reaching 96.3% of the French flag target morphology on this simulation (figure 4*a*). The tissue was observed to spontaneously become stressed several times during its lifetime; these stress increases were not the consequence of any external perturbation, and were due to the intrinsic dynamics of the tissue. The first stress increase, as described above, happened prior to step 90 in order to reach enough of the target morphology to stay alive. We observed (e.g. in the representative individual shown in figure 4*b*) four additional stress increases at steps 285, 322, 395 and 896. At these steps, the intrinsic dynamics of the tissue created deviations from the target morphology that increased the level of stress, after which the tissue corrected the new anatomical trajectory in order to reach homeostasis, at which point stress decreased. Once the tissue reached a morphology compatible with life, stress was reduced to 0. On average for this task, the collective reached 87.7±13% of the target morphology over 20 runs. These deviations represent spontaneous remodelling as no external perturbation has been applied to trigger them. Remodelling first begins after a time window three times greater than the time needed to reach the target morphology during development. In other words, the morphogenesis can be said to have been completed and ceased long before spontaneous morphogenetic activity suddenly resumes. This phenomenon is similar to that described by catastrophe theory [72], and suggests self-induced missing-tissue response (MTR) [73,74], a process similar to the one we observed in the planarian experiments (see §6). In our simulations, a dramatic spike in stress immediately precedes the sharp onset of extensive morphogenetic activity (see electronic supplementary material, figure S1*a*), suggesting that stress drives the morphogenetic changes in the tissue.

Interestingly, the cells had never been evolved over a period of time greater than 100 steps—there was no selection pressure for long-term stability or survival. However, they were able to maintain the tissue more than 10 times longer than their initial developmental period (the only aspect on which they were evolved). We were also surprised to find they also learned allostasis. Following the definition of McEwen & Wingfield [75]: allostasis is the process of maintaining stability (homeostasis) through change in both environmental stimuli and physiological mechanisms. In this simulation, the tissue changed regularly as it tried to get rid of remaining, inappropriately located blue cells; sometimes this remodelling process took the collective a sufficient distance from the French flag target morphology to activate homeostatic mechanisms which then drove it back to normal, allowing long-term maintenance and survival of the tissue.

Thus, we conclude that long-term stable survival does not need to be specifically selected for, as intrinsic dynamics and emergent anatomical regenerative capacity are enough to maintain order through an adult phase. Homeostasis is an active process which is mirrored in this case by the level of stress that increases and decreases with the distance between the current tissue state and the target morphology.

### 5.4. Stress: different use-cases

The morphogenetic process exhibits interesting stress dynamics, consistent with the proposals [7] that homeostatic loops are driven by stress as a reflection of delta from setpoint, and that complex setpoints such as tissue-level morphogenetic patterns could arise from cells sharing stress information to optimize plasticity and coordinate in more complex problem spaces. Thus, we next studied the functional role of stress in the emergent morphogenesis we observed.

#### 5.4.1. Evolution exploits stress as an instructive signal to reach the target morphology

**Figure 5.**
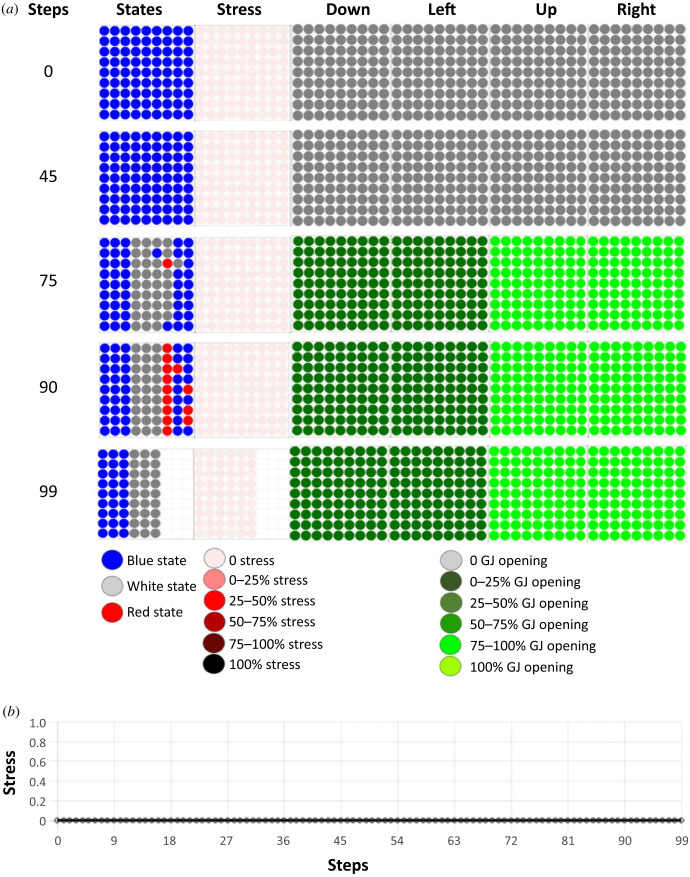
Loss of function experiment for stress: dynamics of resolution of the French flag problem with artificial injection of anxiolytics. (*a*) Time steps of the dynamics of resolution of the French flag problem by the tissue after artificial injection of anxiolytics. Stress was artificially reduced to 0 with simulated anxiolytics. The dynamics of the tissue is represented by five time steps taken at 0, 45, 75, 90 and 99 steps of the dynamics of the tissue. These five time steps are indicated in the leftmost column, and the remaining columns represent (from left to right): the states of the cells in the tissue (blue, white, red), the use of the stress system on a colorimetric scale (the darker the red, the more the system is stressed), and the percentages of open gap junctions in four directions (down, left, up and right), again using a colorimetric scale. Grey represents closed gap junctions and the green scale denotes the range of opening: the darker the green, the smaller the percentage of open gap junctions. The cells behave completely differently in the absence of stress: at step 90, the tissue had not yet reached the French flag configuration and at 99 steps, the cells located on the red stripe died. (*b*) Stress is artificially held steady at 0 with simulated anxiolytics (a loss-of-function experiment for the stress system).

We first sought to determine whether stress was instructive or merely a by-product of the various dynamics. To answer this question, we simulated a loss-of-function of the stress system in the tissue (e.g. as the action of an anxiolytic drug), which forcibly reduced the level of stress to 0 during the whole lifetime of the embryo (figure 5).

The dynamics of the gap junctions were similar in this anxiolytic experiment to what they were when stress was used (figure 2). However, we observed that at 90 steps, the tissue reached only 82.8% of the target morphology. And at 99 steps, the collective of cells corresponding to the location of the red stripe of the French flag dies. The collective achieved 67.3±2.8% of the target morphology over 20 runs.

These experiments reveal that, in the absence of stress, anatomical homeostasis is not reached nor maintained during the lifetime of the cells. By contrast, when stress is available to the cells, the tissue reaches 95.1% of the French flag target morphology at 90 steps and it maintains the morphology after this step.

Thus, we conclude that cells exploit the ability to use the stress mechanism to implement morphogenesis and survival of development.

#### 5.4.2. Stress is instructive but only at particular levels

**Figure 6.**
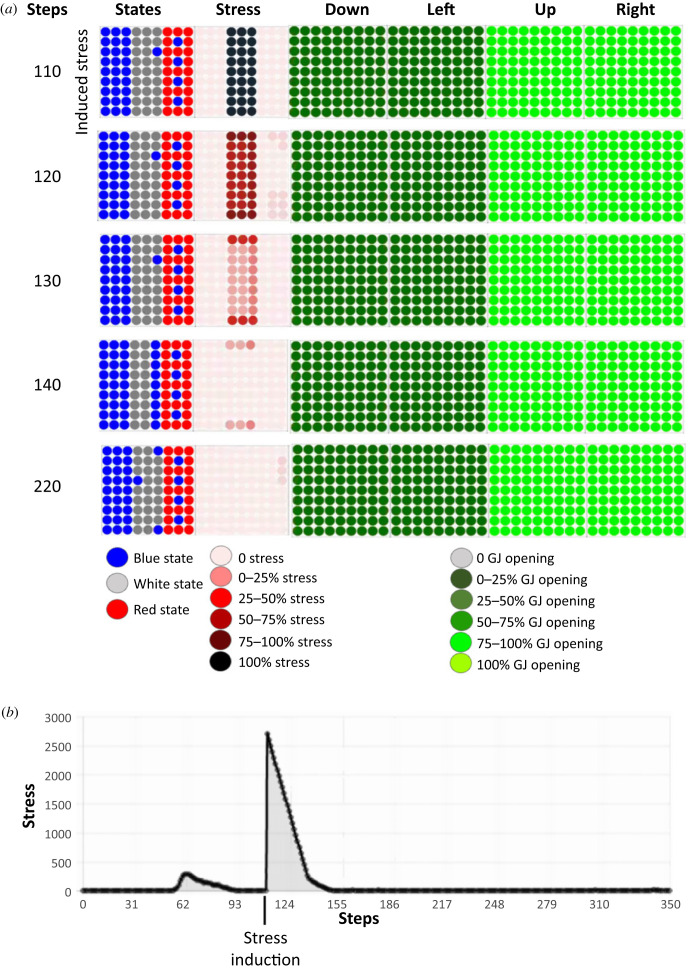
Dynamics of the resolution of the French flag problem after a steep artificial increase in stress. (*a*) Time-lapses of the dynamics of resolution of the French flag problem by the tissue. The dynamics of the tissue is represented by five time steps taken at 110, 120, 130, 140 and 220 steps of the dynamics of the tissue. These five time steps are indicated in the leftmost column, and the remaining columns represent (from left to right): the states of the cells in the tissue (blue, white, red), the use of the stress system on a colorimetric scale (the darker the red, the more the system is stressed), and the percentages of open gap junctions in four directions (down, left, up and right), again using a colorimetric scale. Grey represents closed gap junctions and the green scale denotes the range of opening: the darker the green, the smaller the percentage of open gap junctions. Stress is artificially increased drastically at 110 steps. At 140 steps, a wall of blue cells appears in the white stripe, suggesting that stress is informative but only at particular concentrations. (*b*) Amount of stress in the tissue during its lifetime. The drastic artificially induced stress is seen at 110 steps, after which it decreases linearly until it reaches 0 around step 150.

Having seen the importance of stress mechanisms among cells, we next wanted to know if the stress system featured a window of optimality: is too much stress bad for this process, as it is for real biological systems? Thus, we artificially induced a very high stress (compared to what is needed and used during the French flag resolution) at the locations of the white stripe at 110 steps of the simulation (figure 6). With this excess stress, we simulated an environmental stressor that is not adaptively informative about the current situation of the tissue since it had already reached a version of the French flag compatible with survival (e.g. the cells receive enough energy). Stress decreased immediately without changing the states of the cells. At step 140, a line of blue cells appeared in the white stripe. It seems that stress was only instructive at specific concentrations inside the cells, and then blue cells appeared in the white stripe. Stress transformed the cells close to the borders of the stripes into reservoirs of morphogen molecules, and by accumulating them, they became blue. It seems the cells evolved by making stress instructive only at specific levels as it had no effect during those last 30 steps and it kept decreasing.

We conclude that evolution used stress as a communication system using a particular encoding, in which stress level is instructive and has effects on cell behaviour only at specific concentrations.

#### 5.4.3. Evolution exploits the stress system but anxiolytics do not always have an impact on anatomical homeostasis

Evolution finds more than one way to solve a problem. Here, we analysed the counterexamples—the individuals that can solve the problem without the use of stress. In 10 evolutions that achieved a fitness score superior to 90% on average (over 20 runs) for reaching the French flag, simulations acheived 93±2.6% of target morphology with stress and 91.5±8% when anxiolytics are added. However, 70% of the evolved tissues use the stress system but it is not systematically functional. This high standard deviation for the anxiolytics experiment is explained by the fact that for the majority of the evolved individuals, stress is not fundamental to reach the target morphology. For one evolved tissue, the absence of stress results in embryonic death during development, while for others it has no negative impact. Quantitatively, 1/10 of the evolved tissues used stress as an instructive signal and died when anxiolytics were added. This is true for the evolved tissue in the different experiments above. This ratio is low but, in terms of biological evolution, it would be sufficient to spread into the entire population over time if it increases the overall fitness of the organism during evolution. The other virtual embryos are still able to develop the French flag without stress even if they used it as a result of artificial evolution. Three in 10 of the virtual embryos had better scores on average on the French flag in the presence of anxiolytics, meaning that stress is a noisy signal for them.

**Figure 7.**
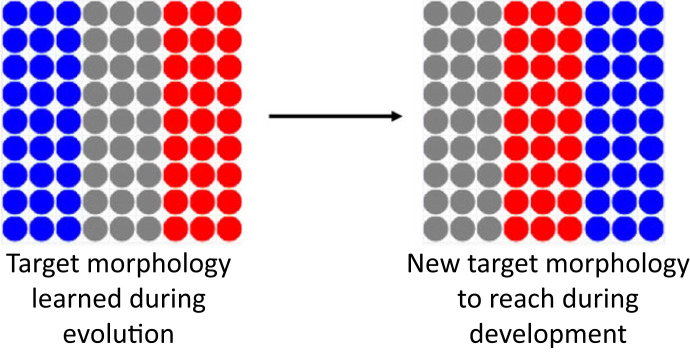
Representation of the target morphology learned during evolution, e.g. the French flag (left) and the new target morphology the tissue has to reach at 60 steps (right).

Why does biological evolution use stress—does the use of the stress system in our simulation increase the adaptive responses of the organisms in more complex scenarios? In order to answer this question, we used the same 10 virtual evolved tissues that achieved a fitness score superior to 90% on average over 20 runs for reaching the French flag and ran an experiment where the target morphology changes during the development phase (at 60 steps; see figure 7) and cells that die are immediately regenerated by white cells. With these conditions, the 10 virtual embryos obtained a mean score of 74±17.4% on the new target morphology. For the one embryo using the stress system as functional, we obtained a mean score of 89.2±4.5% and for the nine others that do not use stress as instructive, the mean was 73±18.6%. We applied a one sample *t*-test to compare these two means and found the difference statistically significant (with *p*-value = 0.0312). Thus, it is possible that stress enables adaptive responses in potentially harmful environments.

In summary, we found that the stress system is not always needed to resolve the French flag problem during development. For this problem, anatomical homeostasis without stress is possible but exploiting spatial propagation of stress information seems to increase adaptive fitness by improving performance in scenarios where the normal process of morphogenesis is impacted by external perturbations. As has been suggested [7], stress is an ideal parameter for evolution to exploit as a representation of the error in homeostatic contexts, and the drive to reduce stress could then implement ‘grow and remodel until complete’ in regenerative settings [7].

### 5.5. Information-theoretic analysis of the dynamics of the anatomical homeostasis

**Figure 8.**
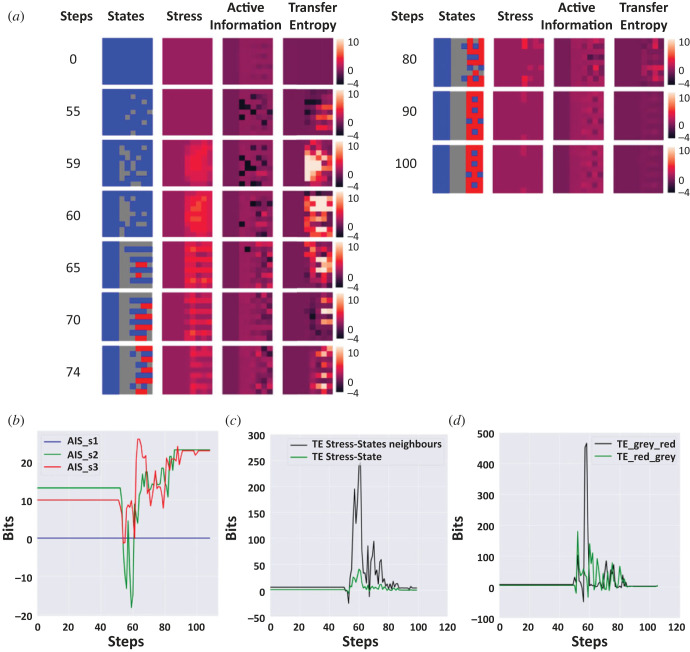
Information-theoretic analysis of tissue dynamics during the resolution of the French flag problem. (*a*) Time steps of the dynamics of resolution of the French flag problem by the tissue. Tissue dynamics are represented by 10 time-lapses taken at 0, 55, 59, 60, 65, 70, 74, 80, 90 and 100 steps. The first column represents these steps, the second column represents the states of the cells in the tissue (blue, white, red), and the third represents the use of the stress system on a red scale. The last two columns represent, respectively, active information and transfer entropy from stress to state of neighbouring cells. (*b*) Representation of the sum of local active information storage (AIS) for each stripe: s1 is the blue stripe (blue), s2 is the white stripe (green), and s3 is the red stripe (red). AIS peaked first for the red stripe. (*c*) Representation of the sum of the averaged local transfer entropy from stress time series to the state time series of the neighbours (black) and the transfer entropy from stress states to the internal states of the cells (green). The transfer entropy was much higher between stress and state of neighbouring cells than between stress and internal state. (*d*) Representation of the transfer entropy from one stripe to another: white to red stripe (black), and red to white (green). The transfer entropy from one stripe to another peaked at different times suggesting that they alternated in driving state changes.

To truly understand how cellular collective behaviour solves morphogenetic problems, and to derive efficient interventions for biomedical contexts, it is essential to understand what the cells are communicating to each other: what information is available to groups of cells? How does its propagation through the tissue enable the cohesion of multiple competent agents into an emergent individual at a higher level of organization that can achieve a morphogenetic goal? Thus, we next applied information-theoretic measures to analyse the dynamics of the tissue during anatomical homeostasis. We computed the local active information and local transfer entropy for the original simulation without any perturbation (figure 2). We also computed the transfer entropy from one stripe to another to understand if one sub-collective could drive the information dynamics and ultimately the anatomical homeostasis (figure 8).

The transfer entropy from stress of one agent to its neighbour started to increase in the tissue at time step 55. Then we wave an important increase at time step 59 in the locations of the white and red stripes. This correlates with the increase of stress in the same spatial locations. The overall transfer entropy was almost 300 bits at this time step (figure 8*c*). Then, the transfer entropy decreased and reached a steady state around 0. The black squares represent negative local transfer entropy, meaning that the cells receive information from the stress that is misleading for predicting their next state. We also compared the transfer entropy from stress of one cell to its own state and found it to be much less important than the transfer entropy from the stress of one cell to the states of its neighbours (figure 8*c*). This suggests the key signalling mode for the stress is paracrine, not autocrine.

The dynamics of the local AIS on the cell states followed different dynamics. When transfer entropy was increasing, we observed several spatially located negative local AIS among the spatial locations of the white and red stripes (see figure 8*a*). At 55, 59 and 60 time steps, the AIS was mainly negative for the white stripe, after which it increased gradually until it reached steady state (figure 8*a*,*b*). For the red stripe, AIS also decreased globally until around step 70 and then we observed an important increase, indicating cells could predict better their future state from the past memory. Concurrently, the stress is decreasing. The local AIS of the blue stripe was 0 throughout as the cells in this region never changed their states. Thus, stress increases concurrently with a decrease in local AIS in the tissue (as seen in figure 8*b*) or, in other words, when the cellular memory does not effectively predict the future. This is compatible with the definition of stressors as unpredictable and/or uncontrollable stimuli [76,77].

We also computed the average local transfer entropy from one stripe to another, from the cells corresponding to the spatial locations of the red stripe to the white and conversely (figure 8*d*). This averaged transfer entropy was 0 at each time step for the blue to red and white stripes and from each of those to the blue. Using a time window of *k* = 4, before 60 steps, we observed a peak of 250 bits of transfer entropy from the red stripe to the white, suggesting the tissue changes were driven by the red stripe. Just after 60 steps, this reversed: transfer entropy from the red stripe to the white stripe decreased and transfer entropy from the white stripe to the red became much higher, with a peak around 450 bits of information suggesting that the white stripe was now driving the change in the states. The ‘organizer’ region (here defined as a region or group of cells in a tissue that can induce and instructively pattern other cells) seemed to change over time, suggesting embryonic regions take turns driving the large-scale process.

**Figure 9.**
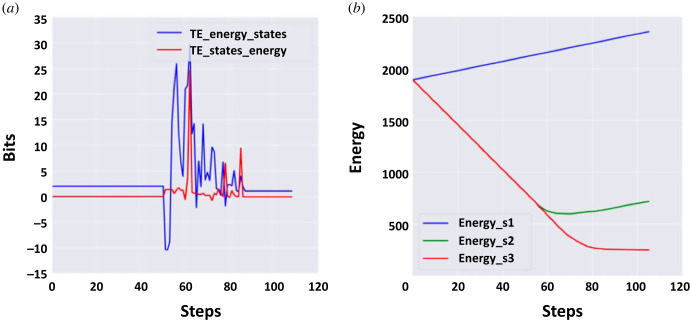
Information-theoretic analysis of multi-scale homeostasis during the resolution of the French flag problem. (*a*) Transfer entropy from energy to states (blue) and from states to energy (red). The first one peaks first, suggesting that initial information flow was primarily from the anatomical homeostatic loop. (*b*) Total energy summed across all cells within each stripe. The blue stripe is represented in blue, the white stripe in green and the red stripe in red. When the French flag configuration was reached, the cells received energy.

Finally, to evaluate the direction of information flow, we looked at local transfer entropy from energy to the state of each individual cell, taking an average across all cells in the whole tissue, as well as the converse—transfer entropy from the cell state to energy (figure 9*a*). The first peak we observed, at around 55 steps, corresponded to the transfer entropy from energy to states, indicating information flow came from the anatomical homeostatic loop. After 60 steps, the two transfer entropies overlapped. At the same time, the average energy of all stripes was either increasing or started to stop decreasing (figure 9*b*). Thus, in our scheme, the information was first driven by the anatomical loop, and the energy needed for single-cell survival was controlled by this loop.

We conclude that stress was very informative in the state changes of direct neighbours of one cell. Stress increased concurrently with a decrease in local AIS in the tissue (as seen in figure 8*b*). This is similar to biological systems where stressors are defined as unpredictable stimuli. In our scheme, evolution used the stress system as a communication system that was activated when memory of past events was not effective for predicting the future. There was a wave of information flow from red to white stripes, and it seems that the larger anatomical homeostasis loop came first in terms of information flow from energy to differentiation state. Taken together, these analyses reveal the dynamics of the information flow in the tissue. We observed an emergent behaviour when the ‘organizer’ region changes over time, stress is used as a communication system with the neighbours, and the higher level of homeostasis (anatomical homeostasis) came first in the dynamics suggesting that top-down information is key to manipulate the dynamics of the tissue.

## 6. Experimental results on repatterning of planaria

The sudden remodelling of cell fates after a long period of apparently quiescent, stable pattern in our model (figure 4) predicts that something similar may occur in biological systems. Might some phenotypes that seem complete in fact need to be followed for much longer to observe the true dynamic? We indeed found this to be the case [78], for planarian flatworms [79] regenerating after exposure to U0126, a blocker of ERK/MAP kinase signalling which plays an important role in many model systems of regeneration [74,80–82]. Several studies have shown that ERK inhibition immediately following amputation leads to the formation of headless animals [74,80,81]. These headless animals that regenerate after ERK inhibition had previously been assumed to have reached a terminal, stable morphology [74]. However, when we monitored them for 18 weeks following ERK inhibition, we observed a remarkable phenotype in which some of the headless animals suddenly began to repattern, with some regaining a wild-type single-headed morphology.

**Figure 10.**
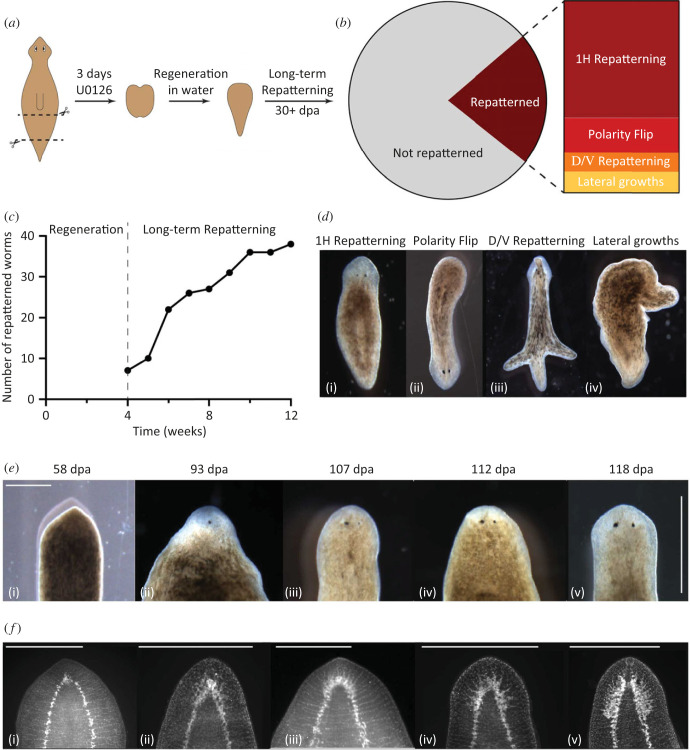
Headless animals can regain normal morphology over long timeframes. (*a*) Illustration of the experimental procedure in which pre-tail fragments were treated for 3 days with U0126 to induce headless animals which were then observed for changes in morphology over long timeframes. (*b*) Out of 181 headless worms tracked individually over time, 22% repatterned, while the remaining 78% did not change their morphology. The repatterned animals fell into four different categories: 61% exhibited single-head (1H) repatterning, 18% polarity flip, 10% dorsal/ventral repatterning and 11% lateral growths. (*c*) Repatterning rate over time showing steady increase in the number of repatterned animals from 4 to 10 weeks after the initial amputation. (*d*) Representative images of the four different repatterning types—(i) 1H repatterning, (ii) Polarity flip, (iii) D/V repatterning (arrow marking dorsal outgrowth) and (iv) Lateral growths. (*e*) Repatterning to regain a single-headed morphology in one animal tracked over time. Scale bar, 500 μm. (*f*) Samples were fixed and stained with synapsin antibody at different stages of repatterning, arranged into a putative neural repatterning timeline. Scale bar, 500 μm.

To quantify this phenomenon, we defined repatterning as any morphology change occurring after four weeks post-cutting. In headless animals observed from when they were cut until their death (*n* = 181), 22% showed some form of sudden repatterning even though regeneration had completed and morphogenesis had ceased weeks prior (figure 10*b*). Importantly, this repatterning occurred without any intervention. The remaining 78% of headless animals did not show any change in morphology except for shrinking in size, as headless animals are unable to feed and therefore allometrically scale down over time [83]. We observed repatterning from four weeks onwards and the majority of repatterning occurred between 4 and 10 weeks (figure 10*c*). After 10 weeks only sporadic new repatterning was observed, although some worms began forming new heads as late as 18 weeks after cutting. This spontaneous repatterning that allows the re-establishment of a normal morphology many weeks after regeneration is completed suggests the existence of tissue processes that operate on a time scale much longer than previously known. Anatomically, the observed repatterning fell into four different categories. In the most common repatterning type (61% of all repatterning worms), normal single-headed wild-type morphology was regained (figure 10*b*,*d*(i)). The second most common phenotype (18%) was a polarity reversal, in which, following fissioning of a headless animal, the posterior blastema formed into a head (figure 10*b*,*d*(ii)). The remaining two categories represent the rare animals that exhibited growths which did not lead to the formation of a head, in the form of either dorsal outgrowths (D/V repatterning, 10%, figure 10*d*(iii)) or lateral outgrowths (11%, figure 10*d*(iv)). When headless animals repatterned to form a new head, the pigmentation of the round anterior end began to lighten and the tissue flattened out, forming a structure resembling a blastema (figure 10*e*). Once the blastema formed, first one eyespot appeared (figure 10*e*(ii)) and then the second one formed with a few days' delay (figure 10*e*(iii)), along with the head reshaping to regain the typical morphology (figure 10*e*(iv)(v)). In addition, animals were stained using synapsin antibodies to visualize regrowth of the underlying brain tissue at intervals during the repatterning process. This showed that early brain repatterning occurred via the formation of a cluster of neural tissue at the anterior rounding of the ventral nerve cord (figure 10*f*(i)). Brain structures formed progressively from there, first in an apparently unorganized manner (figure 10*f*(ii)), before expanding and reforming into a well-organized brain resembling that of a normal animal (figure 10*f*(iii–v)). While the repatterning process resembles normal head regeneration in its progression, the timeframe was distinctly slower than normal head regeneration [84], taking up to 25 days from the first observation of changes at the anterior to a fully formed head.

## 7. Discussion

One of the key open questions in evolutionary developmental biology, as well as basal cognition [8,20], is how single-cell capacities (competency in physiological and metabolic spaces) scale up to enable systems to solve problems in morphogenetic space (anatomical homeostasis), such as axial polarity and body-wide positional information axes. Because intelligence can be defined as competency in navigating arbitrary problem spaces, and all agents are fundamentally composites of other parts, it is important to understand how evolutionary forces and generic dynamics [85–87] work together to scale up collectives and pivot their simple homeostatic properties into more complex capabilities. Here, we presented an evolutionary simulation of the scaling of goals from individual cell-level metabolism to embryo-wide axial positional information. This system is a model of multi-scale homeostasis that illustrates how cells can join into collectives that solve problems in new spaces. We found that evolution was able to use the shared stress system to coordinate across space and time, and developed error-minimization and homeostatic capacities (as demonstrated by robustness to perturbation). The resulting tissue was found to be robust to external perturbation, and use of the stress system was both instructive for initial morphogenesis and necessary for long-term survival.

While maintaining allostasis, the tissue also tried to get rid of the few aberrantly placed blue cells after development, taking the collective temporarily away from the French flag target morphology, to then allow homeostatic mechanisms to drive it back to normal, resulting in long-term maintenance and survival of the tissue. This corresponds in the TAME framework [7] to ‘delayed gratification’—the ability to not get trapped in local minima by temporarily stepping further from the goal in the problem space. This ability to move away from the goal in order to reach a better solution later (following not only minimum-distance paths) is an important capability of some cognitive systems. Degrees of this kind of ‘delayed gratification’ in cybernetic systems enable more sophisticated behaviour, along the continuum from simple ‘roll downhill’ mechanisms to complex problem-solving systems [7]. Interestingly, neither of these things were directly selected for during the evolutionary cycle, revealing how some capabilities of multi-scale homeostatic systems can emerge as a kind of ‘free lunch’, without direct selection pressure [2,88].

Our analysis reveals the emergent functional connections between the single-cell and anatomical homeostatic loops. Each cell has one goal, to survive, and it corresponds to being in the appropriate state to receive energy (with the other members of the collective), e.g. navigating in a one-dimensional metabolic continuous space but by discretizing it: the new emergent problem space solution has three instances (blue, red, white), corresponding to different cell fates along a positional information axis. On the other hand, the collective/tissue has a morphogenetic goal, the French flag, and it is simultaneously navigating a problem space of 3^*n*^ instances (*n*, number of cells).

Our model made a surprising prediction—spontaneous and sudden re-initiation of morphogenetic activity long after patterning has completed. This phenomenon was recapitulated by the results of *in vivo* experiments in planaria. Of course, our model is provisional, and not claimed to definitively capture all of the complexity of the biology, but the fact that it reproduces surprising behaviour not predicted by other known models is reassuring. Such minimal models are an important contribution to the field of molecular biology and genetics, which are awash in enormous datasets of detail and in need of conceptual tools for extracting understandable meso-scale dynamics sufficient to explain the observed biological robustness.

The observation that planarians are capable of recovering a wild-type bodyplan by spontaneously triggering a regeneration-like process, after maintaining a stable abnormal morphology for long periods of time, poses interesting questions about how abnormal morphologies are first maintained and how they are eventually detected to trigger correction. In other organisms, there is some evidence that remodelling of tissues to fit new morphologies can be part of regeneration, such as the remodelling of transplanted tail tissue into a limb in amphibia [89]. The repatterning reported here is distinct from previous studies where it was shown that general injury signals can induce regeneration in headless animals [74], as here we applied no triggering injury of any kind. The newly demonstrated ability to mount a regenerative response without any triggering injury is consistent with the idea that a MTR may not be necessary for regeneration to occur [90] and that subtle internal dynamics may be sufficient to initiate change long after the process appears to have reached a stable point. Our finding that the spontaneous repatterning is slower than normal regeneration is also consistent with other examples of regeneration in the absence of the MTR [90]. The timepoint at which repatterning starts appears to be stochastic within the population. The observation of sudden initiation of repatterning in a seemingly quiescent tissue raises a key question: what process in the tissue serves as a ‘clock’ that enables remodelling at a particular time frame? It must be distinct from the on-going and obvious morphogenetic processes because remodelling initiates suddenly, on a background of constant cell identity distributions.

Recently, Chou *et al.* found a dynamic in bacterial biofilms similar to the clock and wavefront model describing somitogenesis [91]. Interestingly, they observed that nitrogen stress response in the biofilm can form a complex pattern of concentric rings—revealing that spatial distribution of stress responses had occurred already at the time that social microbes emerged. Their results show that the development of bacterial biofilm collectives is driven by an oscillatory process that can locally amplify nitrogen stress responses, such that spores may develop in different areas of the biofilm even under a more abundant nutrient environment (not only in the starved interior of the biofilm). Interestingly, in our model, we observe a similar behaviour with a subtle oscillatory stress response of the cells in different areas of the tissue (figures 1*b* and 8*a*) allowing the formation of the French flag configuration. We found that the pattern generated by the stress response in our simulated tissue defines spatial regions where cells differentiate into the different stripes, a mechanism similar to that seen in the biofilm when various strains of bacteria differentiate into spores. This behaviour is similar to somitogenesis where a homogeneous tissue becomes segmented (transcriptionally and anatomically). In both our simulation and the biofilm, stress seems to play a key role and convey information leading to differentiation of the cells and bacteria similar to the clock and wavefront model. Our system is not similar in every aspect, but they resolve the same problem: how to create spatio-temporal order and to shift from cell-level metabolic goals to the much larger tissue-level morphogenetic goals. Thus, conserved dynamics could be relevant across bacterial, metazoan and *in silico* minimal models of the scaling of spatial patterning from single-cell metabolic and stress dynamics.

The communication system is fundamental for collective intelligence. In our scheme, communication is mediated via gap junctions, a well-known system for coordinating physiological and morphogenetic activity which has also been proposed to be an essential complement to enhancing collectivity [20,41,92]. In our simulation, three types of molecules can be exchanged: the morphogen, the stress molecule and its counterpart, the ‘anxiolytics’. In our simulation, stress plays a key role. Stress is an ambiguous and transdisciplinary concept we used from material science to physiology and ecology [62,93,94], encompassing different meanings: certain stimuli can be stressors; the emergency responses to the stressor is defined as the stress response; and chronic stress is the over-stimulation of the emergency responses [62]. In addition, the definitions of these three concepts are mutually dependent: a stimulus that initiates a stress response is a stressor, but the physiological or behavioural response is considered a stress response if it is initiated in response to a stressor. One solution has been to define stressors as stimuli that disrupt or threaten to disrupt homeostasis [95], but the concept of homeostasis has its own limitations [96]. A more general definition is that stressors are unpredictable and/or uncontrollable stimuli [76,77]. Interestingly, this last definition fits with the emergent use of the stress system in our simulation. Stress increased concurrently with a decrease in local AIS in the tissue as seen in figure 8*b*. Thus, in our scheme, evolution used the communication system as a stress system that was activated when the memory of past events was not effective for predicting the future. This relates to the active inference framework and the free-energy principle, both of which have recently been developed to integrate homeostasis and allostasis [97], as applied to morphogenetic systems [98,99].

Network structure is key to collective intelligence [67,100,101]. It has been shown that flat, fully connected, network structures provide the most efficiency for collectives to resolve a task since that type of structure maximizes the aggregation of information received from members of the collective [100]. However, this kind of network is very costly as it necessitates *n*(*n* − 1) connections and is probably not biologically realistic. Indeed, in most cell networks, cells are only connected to their near neighbours. The stress system can be seen as another communication system and could play a role in changing the network structure to a flat (fully connected) structure, at least for some part of the network, thus facilitating information aggregation (see [102] for a review of non-local cell communication modalities).

One purpose of these kinds of simulations is to develop protocols that could enable robotics and artificial intelligence approaches to benefit from the kind of robustness and problem-solving ability observed in biology. In artificial intelligence, the fact that all intelligences are collective intelligences has not been emphasized [7], but there are recent attempts for swarm robotics and deep learning to develop new methods based on collective intelligence [48,103]. Deep learning has started to integrate the approach with adversarial networks with two networks working together [104]. Artificial collective intelligence is usually studied from the point of view of swarm intelligence or human society [105–107] but morphogenetic systems present several characteristics that swarms lack: a (growing) grid network architecture, the informational wiping property (cells do not know the origin of a given signal), and a different communication system closer to the information processing of a cellular automaton that can only communicate with its direct neighbours. In addition, in this work, we were interested in a reward that has to be explicitly global. In our simulation, individual cells have a high uncertainty on the reward, as it depends on how well the collective is performing. We developed a global mechanism which rewards for complex, non-local dynamics, solving a problem that is also known as ‘credit assignment’ in machine learning. We wanted to address the question of how this long-range reward can drive scale-up of single-cell competencies toward tissue-level goals without having to pre-specify each cell’s correct final state. This is a way of mechanistically linking top-down rewards for system-level performance with the low-level component behaviours, addressing the problem of credit assignment, and is thus a step toward integrating the scaling of cognition findings into artificial intelligence by demonstrating dynamics that can lead to desired emergent properties across scales.

**Figure 11.**
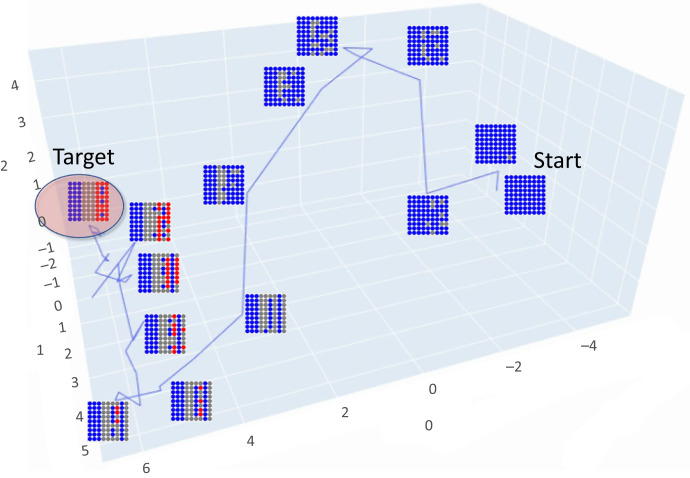
Visualization of the self-organization of development as navigation in morphospace. Three-dimensional PCA projection of the tissue states during resolution of the French flag problem (using results of the first experiment, shown in figure 2, and the PCA explains 81% of the variance in these data). The target represents the region in morphospace that resolves the French flag problem (during development, without any artificial interventions) sufficiently for survival (i.e. a primary body axis that enables high functional fitness).

Lastly, it is interesting to consider the relevance of this system for basal cognition [108,109]. It has been argued that body patterning and behavioural control seem to share a common origin, not only via the mechanisms of ion channels and neurotransmitters, but also via the evolutionary pivot by which biology uses similar processes to solve problems from physiological to morphogenetic and ultimately three-dimensional behavioural spaces [8,9]. Indeed, several somatic tissues exhibit evidence of learning and basal cognition, including cardiac [110], bone [111] and pancreatic tissues [112], in addition to the large literature on learning and decision-making in microbes and other unicellular organisms [113–118]. These capacities are very old [119,120] and the molecular apparatus of higher cognition (ion channels, neurotransmitters and synaptic mechanisms) was already present in our unicellular ancestors [13]. Brains and neurons have been speed-optimized by evolution from other cell types [121]. But somatic cells did not lose their cognitive repertoire and computational capabilities during their transitions to multicellularity or in becoming part of metazoan swarms (bodies): they scaled them to pursue larger anatomical goals [4]. The symmetry between development/regeneration and traditional cognition is not simply an emergent result of hardwired processes, but the emergent result of a very plastic, context-dependent system that achieves invariant patterning outcomes under uncertainty. Biological systems have remarkable capacities to achieve the same morphogenetic outcomes despite a range of perturbations, such as different starting conditions, different numbers and sizes of cells, and various interventions (reviewed in [1,122]). In this work, we simulated the transitions from single-cell homeostasis to anatomical homeostasis using stress, and tested the hypothesis that a minimal evolutionary framework is sufficient to scale small, low-level setpoints of metabolic homeostasis in cells to the larger morphogenetic setpoints of collectives (tissues) (figure 11). These transitions take place in a continuum from body patterning to cognition. We propose that evolution pivoted the collective intelligence of cells during morphogenesis of the body into traditional behavioural intelligence by scaling up the goals at the centre of homeostatic processes. This work is a first step towards a quantitative understanding of the scaling of cognition.

## Data Availability

Raw data are available upon request; software is available at https://github.com/LPioL/scalefreecognition. The data are provided in electronic supplementary material [123].
